# The Impact of Medicaid Expansion on Stage at Diagnosis of Melanoma Patients: A Retrospective Study

**DOI:** 10.3390/cancers17010061

**Published:** 2024-12-28

**Authors:** Ramya Muddasani, Helena T. Wu, Shwe Win, Arya Amini, Badri Modi, Ravi Salgia, Vijay Trisal, Edward W. Wang, Miguel Angel Villalona-Calero, Aaron Chan, Yan Xing

**Affiliations:** 1 City of Hope Comprehensive Cancer Center, Duarte, CA 91010, USA; rmuddasani@coh.org (R.M.); helenawu@uchicago.edu (H.T.W.); swin@coh.org (S.W.); aamini@coh.org (A.A.); bamodi@coh.org (B.M.); rsalgia@coh.org (R.S.); vtrisal@coh.org (V.T.); edwang@coh.org (E.W.W.); aarchan@coh.org (A.C.); 2 Data Science Institute, The University of Chicago, Chicago, IL 60637, USA; 3 Division of Hematology Oncology, University of California, Irvine, CA 92612, USA; mavilla4@uci.edu

**Keywords:** Medicaid, melanoma, Affordable Care Act, immunotherapy, healthcare access, health disparities

## Abstract

Background: This study addresses the lack of research on Medicaid expansion’s impact on melanoma staging, treatment utilization, and outcomes by evaluating its effects under the Affordable Care Act (ACA), particularly focusing on staging at diagnosis, treatment use, and 3-year mortality outcomes. The objective is to determine whether Medicaid expansion led to earlier melanoma diagnosis and improved survival rates among non-elderly adults (ages 40–64) by analyzing data from the National Cancer Database (NCDB). Methods: A total of 12,667 patients, aged 40–64, diagnosed with melanoma from 2010 to 2020 were identified using the NCDB. Difference-in-difference (DID) analysis was performed to analyze tumor staging at presentation between Medicaid expansion states and non-Medicaid expansion states both prior to the expansion and after the expansion. Results: Of the total patients, 2307 were from the pre-expansion time period residing in Medicaid expansion states (MES) and 1804 in non-Medicaid expansion states. In the post-expansion time period there were 5571 residing in the MES and 2985 in the non-MES. DID analysis revealed a decrease in stage IV melanoma at diagnosis (DID −0.222, *p* < 0.001) between MES and non-MES before and after Medicaid expansion. After expansion, in stage IV, the occurrence of primary surgery was 0.42 in non-MES and 0.44 (difference 0.02); DID analysis was not statistically significant. The use of immunotherapy in MES was significantly higher than in non-MES after expansion (*p* < 0.001), although DID analysis did not reveal a statistically significant difference. DID analysis showed a statistically significant decrease in 3-year mortality (DID −0.05, *p* = 0.001) between MES and non-MES before and after Medicaid expansion. Conclusions: This study revealed the positive impact of the ACA’s Medicaid expansion on melanoma stage at presentation, highlighting the importance of public health policies in reducing disparities in mortality rates and early-stage diagnoses. Future research should explore additional barriers to care and evaluate the long-term outcomes of Medicaid expansion to optimize cancer care for vulnerable populations.

## Simple Summary

The Affordable Care Act’s Medicaid expansion aimed to increase healthcare access for low-income individuals by providing Medicaid coverage to more non-elderly adults. This study examines the impact of Medicaid expansion on melanoma diagnosis, treatment, and outcomes, focusing on whether the expansion has led to earlier tumor detection and improved survival rates. By analyzing data from the National Cancer Database, the study reveals that Medicaid expansion is associated with earlier melanoma diagnosis and a decrease in advanced-stage melanoma cases. These findings highlight the importance of public health policies in reducing health disparities and improving cancer care for vulnerable populations.

## 1. Introduction

The Affordable Care Act (ACA), passed in 2010, aimed to enhance healthcare accessibility by increasing the number of patients with health insurance and expanding health insurance coverage for low-income populations [1]. The ACA includes a provision for the Medicaid expansion program, providing states with the option to offer Medicaid coverage to non-elderly adults (ages 19–64) with incomes at or below 138% of the federal poverty level. While six states participated in the Medicaid expansion in 2010, most states implemented the Medicaid expansion in 2014, improving access to care and utilization of healthcare services for previously uninsured, low-income citizens. These individuals are now more likely to receive preventative care, and numerous studies have shown increased cancer screening rates and healthcare utilization in Medicaid expansion states in colorectal, breast, and cervical cancer [2,3,4,5].

Additionally, research studies have indicated that following the expansion, there was increased detection of early-stage disease in patients with various cancers, including breast, colorectal cancer (CRC), head and neck, and lung cancer in Medicaid expansion states and potentially improved survival rates compared to those in non-expansion states [6,7,8]. Melanoma ranks as the fifth most common cancer diagnosis in the United States, with its incidence rising both domestically and globally. From 2006 to 2015, melanoma rates increased in adults at or over 40 years of age by an APC of 1.8%, disproportionately affecting patients of lower socioeconomic status who are at higher risk for delayed diagnosis and worse outcomes due to limited healthcare access [9,10,11,12]. Prior to the ACA’s Medicaid expansion, uninsured individuals were less likely to receive preventative care and timely treatment, resulting in a higher proportion of advanced-stage melanoma cases and worse outcomes in low-income populations [13]. These trends emphasize the need to evaluate the ACA’s impact on addressing these healthcare disparities. For localized melanoma, the standard treatment based on the National Comprehensive Cancer Network (NCCN) guidelines is wide local excision of the primary lesion and sentinel lymph node biopsy (SLNB), if indicated, in patients with T1b or higher stages at high risk of nodal metastases [14]. Following primary surgery, patients may undergo additional treatments with radiation therapy, immunotherapy, targeted therapy, or chemotherapy, as indicated.

The ACA, including the Medicaid expansion provision, was designed to improve overall healthcare accessibility; however, the extent to which Medicaid expansion has impacted tumor staging of melanoma at presentation and melanoma care remains under-explored. Important to acknowledge is the potential heterogeneity within and between Medicaid expansion states (MES) and non-Medicaid expansion states (non-MES). Each state operates within unique political environments, demographic contexts, and healthcare infrastructures that may influence healthcare access and quality of care.

This study aims to evaluate the impact of Medicaid expansion on the primary outcome of interest and melanoma tumor stage, as well as primary surgery, use of immunotherapy, and 3-year mortality, shedding light on the potential benefits of expanded healthcare coverage in low-income communities.

## 2. Materials and Methods

A retrospective cohort study was performed using the National Cancer Database (NCDB), a collaborative project between the American Cancer Society (ACS) and the American College of Surgeons Commission on Cancer (ACS CoC), which is a facility-based, nationwide dataset on more than 70% of new cancer diagnoses in the U.S. and includes collected data on approximately 40 million records from hospital cancer registries in the USA.

**Figure 1:**
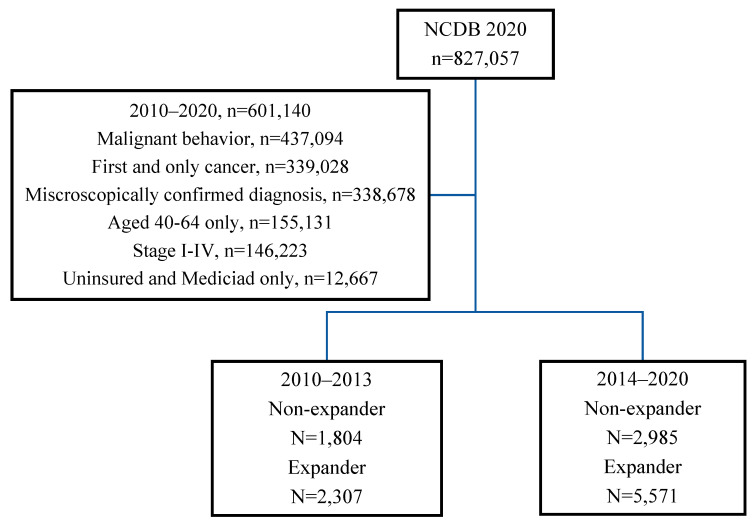
Melanoma cohort—Flow chart of criteria utilized to select patients from NCDB.

The NCDB was used to identify 12,667 non-elderly patients (age range, 40–64) who were newly diagnosed with melanoma from 2010 to 2020. The flow diagram in Figure 1 features the study inclusion and exclusion criteria used to define the study cohort. Patients less than 40 years of age were excluded as the NCDB does not report expansion status information for this age range, and patients older than 64 were excluded as they are eligible for Medicare. Additionally, patients with private insurance and other types of government or unknown primary insurance were not included in the study. The analytic stage is assigned by the NCDB based on the reported pathologic stage; however, if this was unavailable, the clinical stage was used. The sub-stage groups were consolidated into their corresponding general stage designations of I to IV. Patients with unknown analytic stage were excluded from the study. This study analysis was exempted by the Institutional Review Board, and all de-identified data are in compliance with the Health Insurance Portability and Accountability Act.

A ꭕ^2^ test for categorical variables and a *t*-test for means were performed to evaluate the clinical and demographic characteristics of the study population. Year-to-year trend analysis was used to examine trends in melanoma stage and the effect of expansion on insurance status over time between MES and non-MES states from 2010 to 2020.

Difference-in-difference (DID) analysis was performed to analyze tumor staging at presentation between MES and non-MES before the expansion (2010–2013) and after the expansion (2014–2020). Non-expansion states consisted of TN, NC, ID, GA, FL, MO, AL, MS, KS, TX, WI, UT, SC, SD, VA, OK, NE, WY, and ME. The interaction between time periods (2010–2013 and 2014–2020) and expansion status (MES and non-MES) were defined for the DID estimate, and a multivariable linear probability model was used. The multivariable analysis was adjusted for age, sex, race, comorbidity, income, education, population density, facility type, and tumor histology.

The primary outcome of interest was melanoma tumor stage at presentation, and secondary outcomes include primary surgery, use of immunotherapy, and 3-year mortality.

Early-expansion states (WA, CA, NJ, MN, DC, CT) from 2010 to 2013 were excluded from the DID analysis for sensitivity analysis. The parallel trend assumption, a key requirement for DID analysis, was validated by comparing pre-expansion trends in melanoma stage between the exposure group (MES) and control group (non-MES). Visual inspection of the trends in the graph between the two groups before the Medicaid expansion time period between 2010 to 2013 confirms that both groups exhibited similar trajectories in melanoma staging before the implementation of Medicaid expansion, supporting the assumption that any observed post-expansion differences are attributable to the policy.

All tests were two-sided with an alpha of 0.05. Data processing and statistical analyses were performed with SAS software (version 9.1.3; SAS Institute Inc., Cary, NC, USA) and STATA MP (version 13.1; StataCorp, College Station, TX, USA).

## 3. Results

### 3.1. Study Population

In the study population, there were a total of 12,667 melanoma patients (56% male, 95% white) meeting the inclusion criteria. In the pre-expansion time period (2010 to 2013), there were 2307 patients (18.2%) residing in MES and 1804 (14.2%) residing in non-MES. In the post-expansion time period (2014 to 2020), there were 5571 patients (43.9%) residing in MES and 2985 (23.6%) residing in non-MES.

A greater proportion of patients with Medicaid resided in MES compared to non-MES (pre-expansion 51% vs. 32%, post-expansion 82% vs. 40%, *p* < 0.001). Following expansion, the percentage of patients with Medicaid increased by approximately 30% in MES states (Table 1).

Median income was overall lower in non-MES compared to MES both before and after expansion. Before expansion, there were more low-income patients making less than USD 40,227 in non-MES compared to MES (pre-expansion 25% vs. 13%, post-expansion 26% vs. 16%, *p* < 0.001).

Education status was also lower in non-MES compared to MES. In the lowest education group, categorized as 17.6% or more without high school, non-MES had a greater proportion compared to MES (25% vs. 16%, *p* < 0.001), and this was overall unchanged between pre- and post-expansion. As shown in Table 1, MES and non-MES patients have various differences in several demographic characteristics.

### 3.2. Change in Insurance Coverage over Time

In this cohort study, over 80% of patients’ insurance status consisted of private/managed care. In MES, uninsured patients decreased from 3.5% in 2010 to 1.7% in 2020, with the greatest decrease occurring between 2013 to 2014 when more widespread Medicaid expansion took effect. In contrast, in non-MES the percentage of uninsured patients decreased only by 0.6% (6.3% in 2010 to 5.7% in 2020). The proportion of patients with Medicaid increased in MES from 3.4% to 8.9% in the study period. In contrast, patients with Medicaid increased only by 0.4% from 3.5% to 3.9% in non-MES.

**Figure 2:**
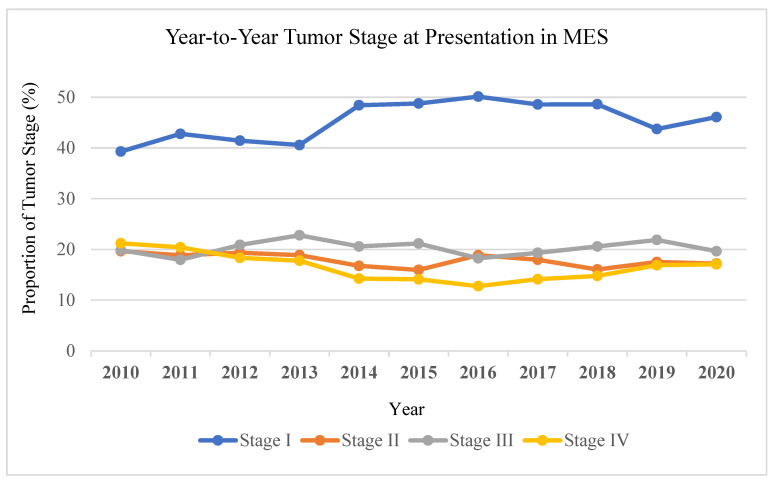
Year-to-year analysis by tumor stage at presentation in MES states.

When accounting for patients without insurance and Medicaid only, there is a clear increase in Medicaid status and, inversely, a decrease in uninsured patients (Figure 2). The proportion of patients with Medicaid increased greatly from 49% in 2010 to 84% in 2020. The proportion of uninsured patients decreased greatly from 51% to 16% in the same period.

### 3.3. Trend Analysis

**Figure 3:**
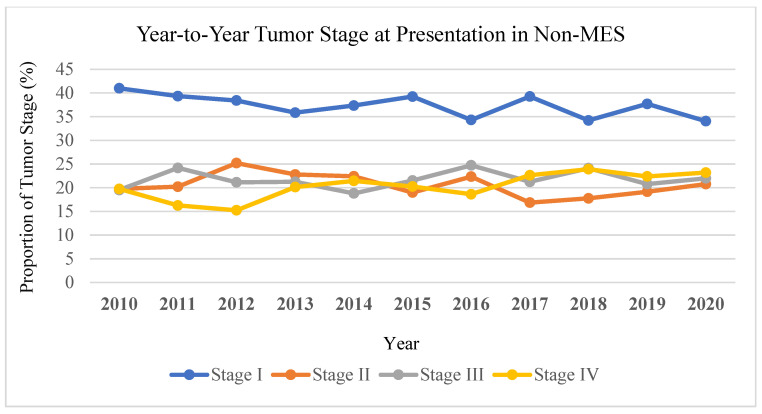
Year-to-year analysis by tumor stage at presentation in non-MES states.

Trend analysis revealed that the proportion of stage IV melanoma at presentation in MES decreased from 21% in 2010 to 17% in 2020. In contrast, stage IV melanoma at presentation in non-MES increased from 20% to 23% over the same period of time (Figure 3). In MES, the proportion of stage I melanoma increased from 39% to 46% from 2010 to 2020, whereas in non-MES, stage I melanoma decreased from 41% to 34%.

### 3.4. DID Analysis

#### 3.4.1. Tumor Stage

Before Medicaid expansion, the adjusted tumor stage of non-MES and MES were approximately the same at 1.71 and 1.77, respectively (difference 0.06). After Medicaid expansion, the adjusted tumor stage in non-MES increased to 1.85, whereas the tumor stage in MES decreased to 1.68 (difference −0.17). DID analysis revealed a statistically significant decrease in stage IV melanoma (DID −0.222, *p* < 0.001) between MES and non-MES before and after Medicaid expansion (Table 2).

#### 3.4.2. Definitive Surgery

Prior to Medicaid expansion, the occurrence of definitive surgery after diagnosis in stage I–III was the same at 0.85 in non-MES and MES. The occurrence of definitive surgery in stage IV was also similar between non-MES and MES at 0.44 and 0.49, respectively, prior to expansion.

After Medicaid expansion, the occurrence of definitive surgery in stage I–III was 0.86 in non-MES and 0.88 in MES (difference 0.02). After expansion, in stage IV, the occurrence of primary surgery was 0.42 in non-MES and 0.44 (difference 0.02). The DID analysis results were not statistically significant for primary surgery between MES and non-MES before and after expansion.

#### 3.4.3. Use of Systemic Immunotherapy

Before Medicaid expansion, the proportion of patients receiving immunotherapy was 0.17 in non-MES and 0.20 in MES (difference 0.03). After expansion, the proportion of patients receiving immunotherapy increased to 0.41 in non-MES and 0.47 in MES (difference 0.06). The results demonstrated that the use of immunotherapy in MES was significantly higher than in non-MES after expansion (*p* < 0.001), although DID analysis did not reveal a statistically significant difference in the use of immunotherapy between MES and non-MES before and after Medicaid expansion.

#### 3.4.4. 3-Year Mortality

The 3-year mortality in non-MES was 0.21, and in MES was 0.24 prior to the Medicaid expansion (difference 0.03). After Medicaid expansion, the 3-year mortality in non-MES decreased to 0.14, and in MES decreased to 0.12 (difference −0.02). DID analysis showed a statistically significant decrease in 3-year mortality (DID −0.05, *p* 0.001) between MES and non-MES before and after Medicaid expansion (Table 2).

## 4. Discussion

In this study involving 12,667 non-elderly melanoma patients, Medicaid expansion was associated with greater insurance coverage in MES compared to non-MES. Overall, the number of uninsured patients decreased by 1.8% in MES, whereas the number of uninsured patients decreased only slightly by 0.6% in non-MES. When only accessing Medicaid and uninsured patients and removing patients with private, government, or unknown insurance status, the percentage of uninsured patients decreased from 51% in 2010 to 16% in 2020.

Although there have been several studies utilizing the NCDB to evaluate the impact of the ACA Medicaid expansion on the staging of various types of cancer, its effect on melanoma is under-studied. This study indicated a positive impact of Medicaid expansion on melanoma stage at presentation. Trend analysis revealed that stage IV melanoma decreased in MES, whereas it increased in non-MES from 2010 to 2020. DID analysis additionally supported these results of decreased tumor stage in MES and increased tumor stage in non-MES. The decrease in tumor stage between MES and non-MES before and after expansion was statistically significant. These findings are in accordance with the findings of prior research studies that measured changes in insurance and cancer staging after Medicaid expansion, with most studies indicating similar results of greater insurance coverage and earlier tumor staging [5,6,7,8]. The results suggest that improved access to healthcare services from the Medicaid expansion can help facilitate the diagnosis of early-stage melanoma for patients of low socioeconomic status due to greater accessibility to healthcare services. Prior literature has indicated that greater screening through regular self-skin examinations and dermatology discussions have the potential to improve melanoma care [15,16]. Additionally, studies have revealed an association between dermatologist skin examinations with earlier melanoma diagnosis, supporting that increased accessibility to healthcare providers and screening examinations with the Medicaid expansion can facilitate earlier diagnosis of melanomas in low-income populations [17,18,19,20]. Overall, the Medicaid expansion appears to provide early diagnosis of melanomas in marginalized communities that are disproportionately affected by melanoma, indicating evidence of the intended benefit of the ACA to provide better care to vulnerable low-income populations.

To our knowledge, our study is the first to examine the association of melanoma patient outcomes with 3-year mortality, use of immunotherapy, and primary surgery after implementation of the ACA’s Medicaid expansion. Our analysis showed that the decrease in 3-year mortality was statistically significant, which suggests that Medicaid expansion positively impacts melanoma patient outcomes. Several studies have indicated that early diagnosis provides more curative treatment options and better prognosis for various cancers, including early-stage melanoma, which is concordant with this study’s findings in decreased 3-year mortality [21,22,23,24,25]. These findings suggest that Medicaid expansion has the potential to improve the outcomes of melanoma patients by promoting earlier diagnosis.

The use of immunotherapy in MES was significantly higher than in non-MES after expansion (47% vs. 41%, *p* < 0.001); however, DID analysis did not show a statistically significant difference in the use of immunotherapy in MES and non-MES before and after expansion. There was also no association of significant difference in primary surgery noted in the DID analysis. The lack of statistically significant difference in the use of immunotherapy and primary surgery can be attributed to systemic barriers such as socioeconomic constraints persisting even after Medicaid expansion, inadequate health literacy, or transportation challenges [26,27]. Moreover, the capacity of healthcare facilities and availability of specialists to perform melanoma surgeries or provide immunotherapy may not have increased proportionally with the expanded Medicaid coverage, which may contribute to the lack of significance in the use of immunotherapy and primary surgery in our results. Regional disparities in healthcare infrastructure could exacerbate these issues, limiting access to advanced therapies and timely surgical interventions. Further research should assess and address the geographic distribution of melanoma care providers and evaluate systemic factors contributing to treatment disparities [28,29]. Lastly, after expansion, tumor staging may have changed where immunotherapy or surgery is not indicated, contributing to the lack of significance in immunotherapy and primary surgery before and after Medicaid expansion. Further investigation is required to evaluate the factors impacting the care of melanoma after Medicaid expansion.

Several limitations should be considered in this study. Firstly, this was a retrospective observational study. Although the DID model was adjusted for several confounders, all potential confounding variables could not be controlled, which may affect the observed associations between variables and outcomes in undefined ways. The DID model may not fully account for concurrent health policies or regional variations in healthcare infrastructure. As the NCDB is facility-based and only includes data from accredited cancer centers, it may not be representative of the entire population treated in non-accredited or smaller hospitals; thus, the prevalence or incidence rates of the general population cannot be determined. The NCDB also does not monitor continuity or disruption of insurance coverage; therefore, the effects of insurance interruption on melanoma stage at presentation cannot be assessed. Additionally, the effects of Medicaid expansion on the young adult population are unknown as the NCDB did not provide information on the expansion status of patients less than 40 years old. It is also important to consider other healthcare policies enacted during the study period and their potential impact on melanoma tumor stage and care. Policies mandating private insurance carriers to fully cover preventive services or various state-specific policies may have contributed to the earlier melanoma diagnoses observed in this study [30,31]. Differences in healthcare workforce distribution, particularly the availability of dermatologists and oncology specialists, may have influenced melanoma care access and outcomes independent of Medicaid expansion [26,32]. Lastly, another limitation of the study is the racial distribution of the study population, in which 96% of participants are white. This lack of diversity limits the generalizability of these findings to other racial and ethnic groups. Melanoma incidence, access to care, and outcomes may differ substantially among non-white populations. Future research may focus on more racially diverse cohorts to determine whether Medicaid expansion has a similar impact on melanoma staging and outcomes in these populations [33]. While these factors cannot be controlled, it is unlikely that they are solely responsible for the melanoma staging trends and should not deter from the major findings of this study.

## 5. Conclusions

This study reveals the positive impact of the ACA’s Medicaid expansion on melanoma stage at presentation and suggests improved access to healthcare services can facilitate the diagnosis of early-stage melanoma and decrease the mortality rate. The findings illustrate the impact of public health policies in addressing the health disparities in vulnerable low socioeconomic populations through greater accessibility to healthcare services and increased insurance coverage. Despite these findings, there are still 16% of patients without insurance in 2020, indicating that continued effort is needed to improve the outcomes of these uninsured patients. Policymakers and healthcare providers should continue efforts to expand healthcare access. Innovative strategies, such as telehealth initiatives, community-based screening programs, and targeted health education campaigns, have the potential to overcome barriers to care and ensure early melanoma detection and treatment for vulnerable populations. Addressing these gaps will be essential to achieving equitable cancer care outcomes.

## Figures and Tables

**Table 1:** Patient clinical and demographic characteristics.

Characteristic	Pre-Expansion (2010–2013) N = 4111			Post-Expansion (2014–2020) N = 5571		
	Non-MES	MES	*p* Value	Non-MES	MES	*p* Value
	N = 1804	N = 2307		N = 2985	N = 5571	
Age at diagnosis (years)—no. (%)			*p* = 0.09			*p* = 0.004
40–49 (0–29 censored)	654 (36)	777 (34)		959 (32)	1622 (29)	
50–64	1150 (64)	1530 (66)		2026 (68)	3949 (71)	
Sex—no. (%)			*p* = 0.80			*p* = 0.009
Male	1039 (58)	1338 (58)		1696 (57)	3002 (54)	
Female	765 (42)	969 (42)		1289 (43)	2569 (46)	
Race—no. (%)			*p* = 0.29			*p* = 1.02
White	1739 (96)	2209 (96)		2858 (95.7)	5290 (95)	
Others	65 (4)	98 (4)		127 (4.3)	281 (5)	
Insurance—no. (%)			*p* < 0.001			*p* < 0.001
Uninsured	1227 (68)	1132 (49)		1804 (60)	991 (18)	
Medicaid	577 (32)	1175 (51)		1181 (40)	4580 (82)	
Charleson-Deyo comorbidity index—no. (%)			*p* = 0.01			*p* = 0.1
0	1468 (82)	1960 (85)		2442 (82)	4647 (83)	
1	257 (14)	258 (11)		373 (13)	664 (12)	
2	55 (3)	56 (3)		103 (3)	145 (3)	
3+	24 (1)	33 (1)		67 (2)	115 (2)	
Median household income—no. (%)			*p* < 0.001			*p* < 0.001
<USD 40,227	452 (25)	300 (13)		748 (26)	874 (16)	
USD 40,227–USD 50,353	523 (29)	435 (19)		762 (28)	1241 (22)	
USD 50,354–USD 63,332	338 (19)	532 (23)		594 (21)	1441 (26)	
>USD 63,332	248 (14)	693 (30)		468 (12)	1074 (19)	
N/A	243 (13)	347 (15)		412 (13)	941 (17)	
Education % without high school—no. (%)			*p* < 0.001			*p* < 0.001
17.6% or more	453 (25)	375 (16)		760 (26)	874 (16)	
10.9–17.5%	545 (30)	532 (23)		845 (28)	1241 (22)	
6.3–10.8%	372 (21)	590 (26)		615 (21)	1441 (26)	
<6.3%	52 (11)	467 (20)		363 (12)	1074 (19)	
N/A	36 (13)	343 (15)		402 (13)	941 (17)	
Population density—no. (%)			*p* < 0.001			*p* < 0.001
Metro	1358 (75)	1885 (82)		2258 (76)	4404 (79)	
Urban	358 (20)	334 (14)		586 (20)	998 (18)	
Rural	52 (3)	47 (2)		79 (2)	90 (2)	
N/A	36 (2)	41 (2)		62 (2)	79 (1)	
Facility type—no. (%)			*p* < 0.001			*p* < 0.001
Community	102 (5)	167 (7)		131 (4)	424 (7)	
Comprehensive	553 (31)	540 (23)		1000 (33)	1374 (25)	
Academic/research	828 (46)	1213 (53)		1362 (46)	2897 (52)	
Integrated network	321 (18)	387 (17)		492 (17)	876 (16)	
Tumor stage—no. (%)			*p* = 0.049			*p* < 0.001
1	696 (38)	947 (41)		1093 (36)	2661 (48)	
2	399 (22)	442 (19)		587 (20)	968 (17)	
3	389 (22)	472 (21)		652 (22)	1125 (20)	
4	320 (18)	446 (19)		653 (22)	827 (15)	

**Table 2:** Difference-in-difference (DID) analysis by tumor stage.

Models	2010–2013			2014–2020			DID (*p*)
	Non-MES	MES	Difference (*p*)	Non-MES	MES	Difference (*p*)	
Tumor stage							
Unadjusted	2.19	2.18	−0.004 (0.92)	2.29	2.02	−0.27 (<0.001)	−0.27 (<0.001)
Adjusted *	1.71	1.77	0.06 (0.08)	1.85	1.68	−0.17 (<0.001)	−0.22 (<0.001)
Definitive surgery							
Stage I–III							
Unadjusted	0.87	0.89	0.02 (0.06)	0.89	0.92	0.03 (<0.001)	0.012 (0.37)
Adjusted *	0.85	0.86	0.01 (0.28)	0.86	0.88	0.02 (0.002)	0.013 (0.33)
Immunotherapy (stage III and IV only)							
Unadjusted	0.20	0.23	0.03 (0.27)	0.43	0.50	0.06 (<0.001)	0.04 (0.23)
Adjusted *	0.17	0.20	0.03 (0.29)	0.41	0.47	0.06 (<0.001)	0.04 (0.20)
3-year mortality							
Unadjusted	0.27	0.28	0.01 (0.27)	0.19	0.15	−0.04 (<0.001)	−0.06 (<0.001)
Adjusted *	0.21	0.24	0.03 (0.02)	0.14	0.12	−0.02 (0.03)	−0.06 (0.001)

* Adjusted for age, sex, race, comorbidity, income, education, population density, facility type, and tumor histology in the multivariate model.

## Data Availability

All data used in this study were obtained from the NCDB.
